# Combination therapy with moderate-intensity statins and ezetimibe and risk of incident PCI/CABG in atherosclerotic cardiovascular disease: a propensity-matched cohort study

**DOI:** 10.1016/j.lanwpc.2026.101895

**Published:** 2026-06-05

**Authors:** Haonan He, Junhan Tang, Vincent Ka Chun Yan, Joseph Edgar Blais, Chun-Ka Wong, Mark Tsz Kin Tam, Hei Hang Edmund Yiu, Hung-Fat Tse, Esther Wai Yin Chan

**Affiliations:** a Centre for Safe Medication Practice and Research, Department of Pharmacology and Pharmacy, Li Ka Shing Faculty of Medicine, The University of Hong Kong, Hong Kong SAR, China; b Department of Medicine, School of Clinical Medicine, Li Ka Shing Faculty of Medicine, The University of Hong Kong, Hong Kong SAR, China; c Cardiac and Vascular Centre, The University of Hong Kong-Shenzhen Hospital, Shenzhen, China; d Department of Medicine and Therapeutics, The Chinese University of Hong Kong, Hong Kong SAR, China; e State Key Laboratory of Pharmaceutical Biotechnology, Li Ka Shing Faculty of Medicine, The University of Hong Kong, Hong Kong SAR, China; f Centre for Regenerative Medicine and Health, Hong Kong Institute of Science & Innovation, Chinese Academy of Sciences, Hong Kong SAR, China; g Hong Kong-Guangdong Stem Cell and Regenerative Medicine Research Centre, The University of Hong Kong and Guangzhou Institutes of Biomedicine and Health, Hong Kong SAR, China; h Centre for Stem Cell Translational Biology, The University of Hong Kong, Hong Kong SAR, China; i Advanced Biomedical Instrumentation Centre, Hong Kong SAR, China; j Accident and Emergency Department, Queen Mary Hospital, Pok Fu Lam, Hong Kong SAR, China; k The University of Hong Kong Shenzhen Institute of Research and Innovation, Shenzhen, China; l Centre for Health AI Research and Translation, Li Ka Shing Faculty of Medicine, The University of Hong Kong, Hong Kong SAR, China

**Keywords:** Coronary revascularisation, Statin, Ezetimibe, LDL-C reduction

## Abstract

**Background:**

Combination therapy with moderate-intensity statins and ezetimibe has demonstrated advantages over high-intensity statin monotherapy for several cardiovascular outcomes. However, its effect on the risk of incident percutaneous coronary intervention (PCI) and coronary artery bypass grafting (CABG) remains unclear. This study aimed to compare the risk of PCI/CABG between combination therapy and high-intensity statin monotherapy among patients with atherosclerotic cardiovascular disease (ASCVD) initially receiving moderate-intensity statin therapy.

**Methods:**

Patients with ASCVD receiving moderate-intensity statin therapy between 2014 and 2023 were identified from the Clinical Data Analysis and Reporting System in Hong Kong. Propensity score matching was used to balance baseline characteristics between groups. Cox proportional hazards regression was used to evaluate the association between the combination therapy versus high-intensity statin monotherapy and incident PCI/CABG.

**Findings:**

After propensity score matching, 1,452 patients receiving the combination therapy and 5,808 patients with the high-intensity statin monotherapy were included. Compared with the high-intensity statin monotherapy, the combination therapy was associated with a significantly lower risk of PCI/CABG (HR: 0.57, 95% CI: 0.36, 0.91). Additionally, those switching to combination therapy had a greater reduction in LDL-C level (absolute change between groups: −0.29 mmol/L, 95% CI: −0.40, −0.19; relative change between groups: −10.6%, 95% CI: −14.1%, −7.2%).

**Interpretation:**

Clinicians may consider the initiation of combination therapy to personalize treatment strategies and improve cardiovascular outcomes.

**Funding:**

No funding has been provided for this research.

**Research in context**

**Evidence before this study**

Current guidelines recommend high-intensity statin therapy for patients with atherosclerotic cardiovascular disease (ASCVD). However, due to concerns about statin intolerance and adverse effects, high-intensity statin therapy remains underutilised in clinical practice. Recently, several studies have compared the effectiveness of high-intensity statin therapy and combination of moderate intensity statin plus ezetimibe, which suggested the benefits of combination therapy on preventing cardiovascular events. We searched PubMED for peer reviewed papers published from inception to 20 February 2026 for all studies reported, using the search terms “statin” (and equivalents), “ezetimibe” (and equivalents), “and “coronary revascularisation”. We found that previous studies had seldom used moderate-intensity statin therapy as the start of lipid-lowering therapy among patients with ASCVD. Additionally, previous studies did not focus on ASCVD patients without history of coronary revascularisation. Given concerns about statin intolerance and side effects associated with high-dose therapy, especially among Asian populations, clinicians are more likely to prescribe moderate-intensity statins initially and escalate treatment as needed. Therefore, it is important to further investigate optimal timing, patient selection, and clinical scenarios targeted for combination therapy.

**Added value of this study**

To our knowledge, this is the first observational study using real-world data to evaluate the effectiveness of combination therapy on PCI/CABG incidence and LDL-C reduction in Chinese patients with ASCVD receiving moderate-intensity statin therapy. For the Asian population, especially Chinese population, who are at higher risk of using high-intensity statin, we found the combination therapy decreased the risk of incident PCI and CABG. Additionally, we measured the change in LCL-C level during the period of receiving combination therapy, which showed an additional reduction in both compared to the use of high-intensity statins, with a higher ratio of achieving the target LDL-C level.

**Implications of all the available evidence**

Using real-world clinical data, our study supports the potential early benefits of using the combination therapy of moderate-intensity statins and ezetimibe in high-risk Asian patients with ASCVD. This approach may reduce the risk of initial PCI/CABG and further lower the LDL-C level. Clinicians may consider the initiation of combination therapy to personalize treatment strategies and improve cardiovascular outcomes.

## Introduction

Atherosclerotic cardiovascular disease (ASCVD) is one of the leading causes for cardiovascular mortality worldwide.^1^, ^2^, ^3^ According to the previous study, over 60% of patients with ASCVD underwent revascularisation procedures after their diagnosis.^4^ Revascularisation procedures, including percutaneous coronary intervention (PCI) and coronary artery bypass grafting (CABG), are typically reserved for patients with advanced or complex ASCVD. They are generally indicated for individuals experiencing persistent angina despite optimal medical therapy, having acute coronary syndromes, having extensive coronary artery diseases that pose a high risk for myocardial infarction or death.^5^^,^^6^ It is recognized that the need for initial revascularisation reflects advanced progression of ASCVD beyond the therapeutic capacity of sole pharmacological management.^7^, ^8^, ^9^, ^10^ Although the current medications for ASCVD cannot replace the necessity of PCI and CABG, various drug therapies may delay the need for revascularisation, which is integral to ASCVD management planning.

Lipid-lowering therapy has become a mainstream treatment in ASCVD management through reducing the level of low-density lipoprotein cholesterol (LDL-C),^11^^,^^12^ where statins are the cornerstone. As the cornerstone of lipid-lowering therapy, high-intensity statin treatment can achieve reductions of more than 50% in LDL-C level, recommended to use among patients with established ASCVD.^13^ Ezetimibe is a lipid-lowering drug that can be added to statin monotherapy when high-intensity statin monotherapy is not tolerated or LDL-C level is not adequately controlled, and the use of combination therapy has been a topic of discussion in recent years.^14^, ^15^, ^16^ The IMPROVE-IT trial was the first randomised controlled trial to assess the effectiveness of adding ezetimibe to statin monotherapy. It demonstrated that combining ezetimibe with statins provided additional LDL-C lowering and improved cardiovascular outcomes.^17^ Additionally, the RACING trial compared the efficacy of high-intensity statin monotherapy versus moderate-intensity statin combined with ezetimibe, and found that the combination therapy was non-inferior to high-intensity statin therapy in cardiovascular death, major adverse cardiovascular events (MACE) and non-fatal stroke, with a greater LDL-C level reduction and better adherence.^16^ Notably, a large nationwide cohort study indicated that the combination therapy might reduce the risk of subsequent coronary artery revascularization compared with high-intensity statin monotherapy among patients with a history of PCI.^15^

The current guidelines recommend that patients with acute coronary syndromes (ACS) initiate a combination of high-intensity statins and ezetimibe or other lipid-lowering agents, emphasizing the superiority of combination therapy over statin monotherapy.^18^ These findings suggest that adding ezetimibe to lipid management may provide additional benefits for patients with ASCVD. Another study indicated that,^19^ compared to direct prescription of high-intensity statin therapy, alternative strategies, such as combining moderate-intensity statins with ezetimibe or titrating statin doses to achieve target LDL-C level might offer advantages in reducing medication discontinuation and dose reduction. Importantly, these strategies did not result in significant differences in the composite outcomes of all-cause mortality, myocardial infarction, stroke, or revascularisation. Notably, previous research compared the effectiveness of combining moderate-intensity statins with ezetimibe and high-intensity statin therapy in lowering the risk of myocardial infarction and recurrent coronary revascularisation among patients who underwent PCI. However, it remains unclear whether, in patients with ASCVD without a history of revascularisation, such combination therapy can still reduce the risk of future coronary revascularisation. For patients who have not undergone revascularisation, delaying the need for such procedures is crucial, as it can reduce downstream complications, the requirement for dual antiplatelet therapy (DAPT), and the overall healthcare burden and costs. Additionally, previous studies have less chosen moderate-intensity statin therapy as the start of lipid-lowering therapy among patients with ASCVD, and further traced different lipid-lowering therapies of higher intensity. Given concerns about statin intolerance and side effects associated with high-dose therapy,^20^, ^21^, ^22^, ^23^ especially among Asian populations, clinicians are more likely to prescribe moderate-intensity statins initially and escalate treatment as needed. This approach underscores the importance of individualized therapy tailored to patient tolerance and risk profiles.

Using an electronic medical records database from Hong Kong, this study aimed to follow the possible treatment strategy in Asian clinical practice, comparing the effectiveness between switching to use high-intensity statin and adding ezetimibe on the risk of incident PCI/CABG, among patients with ASCVD who were prescribed moderate-intensity statins. This study also examined the changes in LDL-C level during the follow-up to provide a more comprehensive assessment of disease progression.

## Methods

### Data sources

This study used data from electronic health records extracted from the Clinical Data Analysis and Reporting System (CDARS) of the Hong Kong Hospital Authority (HA). HA functions as the sole publicly funded healthcare provider in Hong Kong, and serves a population exceeding 7 million individuals. HA administers a network consisting of 43 public hospitals and affiliated institutions, along with 49 specialist outpatient clinics and 74 general outpatient clinics.^24^ CDARS aggregates comprehensive electronic health records, including diagnostic codes, medication prescriptions, clinical procedures, hospital admissions and discharges, as well as laboratory test results. CDARS have been extensively used to generate high-quality research outputs.^25^, ^26^, ^27^ The linkage of electronic health records within CDARS is facilitated through the use of anonymised, patient-specific unique identifiers, ensuring data integrity and confidentiality. In this study, demographic (age, and sex), diagnosis records, prescription records, procedure records, physical examination records (body mass index), and laboratory test records (lipid profiles) were retrieved from CDARS.

### Study design

This retrospective cohort study included individuals from CDARS who had incident ASCVD (identified by International Classification of Diseases, Ninth Revision [ICD-9], Supplementary Table S1) between January 1, 2014, and December 31, 2023. Patients were excluded if their first PCI/CABG was earlier or equal to the date of the first diagnosis of ASCVD.

**Fig. 1:**
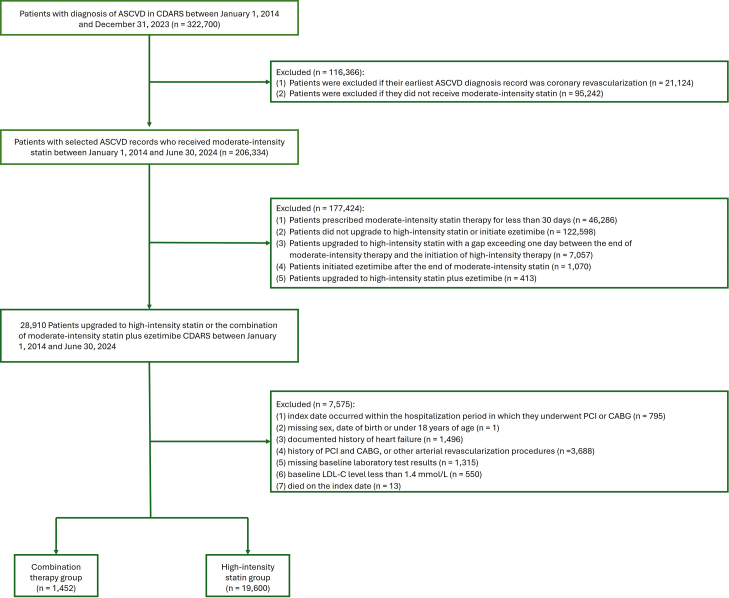
Cohort selection for the analysis.

Among the included patients with ASCVD, the prescription records of statins and ezetimibe from January 1, 2014, to June 30, 2024, were retrieved from CDARS (Supplementary Fig. S1). The moderate-intensity statin therapy was defined in accordance with the American College of Cardiology/American Heart Association guideline (Supplementary Table S3). Patients were further excluded if their prescription period for moderate-intensity statin was shorter than 30 days after the diagnosis of ASCVD. According to the change of lipid-lowering medication until baseline, the eligible moderate-intensity statin users were classified into two groups: (1) the high-intensity statin therapy group, and (2) the combination therapy group, with added medication of ezetimibe (regardless of dosage). In the high-intensity statin therapy group, patients whose starting prescription date of high-intensity statin was more than one day after the end of moderate-intensity statin prescription were excluded. The index date for the high-intensity statin therapy group was defined as the date of starting to use high-intensity statin, while the index date for the combination therapy group was the starting prescription date of ezetimibe. The final sample was established after the further exclusion using the following criteria: (1) index date occurred within the same hospitalization episode in which they underwent PCI or CABG; (2) missing sex, date of birth or under 18 years of age; (3) documented history of heart failure (identified with ICD-9 codes: 428); (4) history of PCI and CABG, or other arterial revascularization procedures (identified with ICD-9 procedure codes: 36); (5) missing baseline laboratory test results (without laboratory test results within 180 days before the index date); (6) baseline LDL-C level less than 1.4 mmol/L; (7) died on the index date (Fig. 1).

### Outcomes and covariates

During the follow-up, the primary outcome was the incidence of PCI/CABG (identified with ICD-9 procedure codes: 36.01, 36.02, 36.05, 36.06, 36.07, 36.1, 36.2). The secondary outcome was a composite outcome of cardiovascular death, myocardial infarction, stroke and PCI/CABG (major adverse cardiovascular events [MACE]) (Supplementary Tables S1 and S2). Cardiovascular death was defined as death with a primarily cardiovascular cause. The changes in LDL-C level were also measured. Two LDL-C targets were set at 1.8 mmol/L, and 1.4 mmol/L. Achievement of each LDL-C target was defined as having at least one LDL-C measurement during follow-up below the corresponding threshold of 1.8 mmol/L or 1.4 mmol/L, respectively. Censoring occurred at the earliest of the following events: (1) initiation of ezetimibe among patients in the high-intensity therapy group; (2) initiation of high-intensity statin therapy among patients in the combination therapy group; (3) discontinuation of treatment (defined as a gap exceeding 90 days between two consecutive prescription records); (4) death; (5) two years after the index date; (6) the end of the study period (December 31, 2024).

Baseline covariates included demographics (age, and sex), calendar year of index date, duration of moderate-intensity statin therapy, comorbidities (including obesity, hypertension, diabetes, liver diseases, chronic kidney diseases, ischemic heart disease, peripheral artery disease and stroke before the index date), medications (including prescription records of antiplatelet, beta blocker, calcium channel blocker, and renin-angiotensin-aldosterone system [RAS] inhibitors within 90 days before the index date), and laboratory test results (including latest LDL-C, HDL-C [high-density lipoprotein cholesterol] and TG [triglycerides] results within 180 days before the index date). Baseline comorbidities and medications were retrieved from the CDARS, using the ICD-9 codes and the BNF codes, respectively (Supplementary Table S4). Notably, obesity was identified based on ICD-9 codes and latest body mass index (BMI) physical examination records within 365 days before the index date, defined as a BMI ≥ 30 kg/m^2^.

### Statistical analysis

Propensity score matching was used to achieve sufficient balance for all baseline covariates. Continuous variables were described as mean (standard deviation [SD]) and categorical variables were reported as frequencies (percentages). Patients in the combination therapy group and the high-intensity statin therapy group were matched at a 1:4 ratio using the nearest-neighbour matching algorithm, and the two groups were considered to be similar if the standardised mean difference (SMD) of the covariates was less than 0.1.^28^^,^^29^

In the matched cohort, the Kaplan–Meier approach was used to describe the cumulative incidence of PCI/CABG and MACE within the two years follow-up, respectively. Incidence rates of PCI/CABG and MACE were calculated for each group. Cox proportional hazards regression models were applied to assess the associations between combination therapy versus high-intensity statin therapy and the risks of incident PCI/CABG and MACE, respectively. Two-sided tests were used, with a significant level of less than 0.05.

During the two-year follow-up period, five subintervals were defined to evaluate changes in LDL-C level: 30–90 days/90–180 days/180–365 days/365–545 days/545–730 days after the index date. In the matched cohort, patients were further excluded if they had no LDL-C test record during the follow-up. Within each subinterval, the mean absolute and relative changes in LDL-C were calculated as the difference between the earliest available laboratory test result and the baseline LDL-C level. The percentages of patients whose LDL-C level was reduced to guideline-recommended target level (1.8/1.4 mmol/L) were calculated separately. A Student’s t test with a two-sided significance level of 0.05 was used to compare the inter-group average changes for LDL-C level across all subintervals in the 2-year follow-up. Chi-squared tests were applied to compare the inter-group percentages of patients whose LDL-C level was reduced to the guideline-recommended target levels (1.8/1.4 mmol/L).

Sensitivity analyses were conducted using two relaxed gap definitions (7 days and 14 days) between the end of moderate-intensity statin therapy and the initiation of high-intensity statin therapy as exclusion criteria, to assess the robustness of the association between combination therapy and incident PCI/CABG during follow-up. Additionally, subgroup analysis was conducted. In the matched cohort, patients were further excluded if their baseline LDL-C level was higher than 2.6 mmol/L, to assess the comparative effectiveness of combination therapy and high-intensity statin therapy. All statistical analyses were conducted in R 4.4. Results were cross-checked by JT for quality assurance.

### Ethics approval

This study was approved by the Central Institutional Review Board of Hospital Authority (Approval number: CIRB-2025-400-3), Institutional Review Board of The University of Hong Kong/Hospital Authority Hong Kong West Cluster (Approval number: UW 25-594), and Joint Chinese University of Hong Kong-New Territories East Cluster Clinical Research Ethics Committee (Approval number: 2025.815), with a waiver of informed consent because of retrospective analysis of de-identified data. This study followed the declaration of Helsinki.

### Role of the funding source

No funding has been provided for this research.

## Results

### Baseline characteristics

**Fig. 2:**
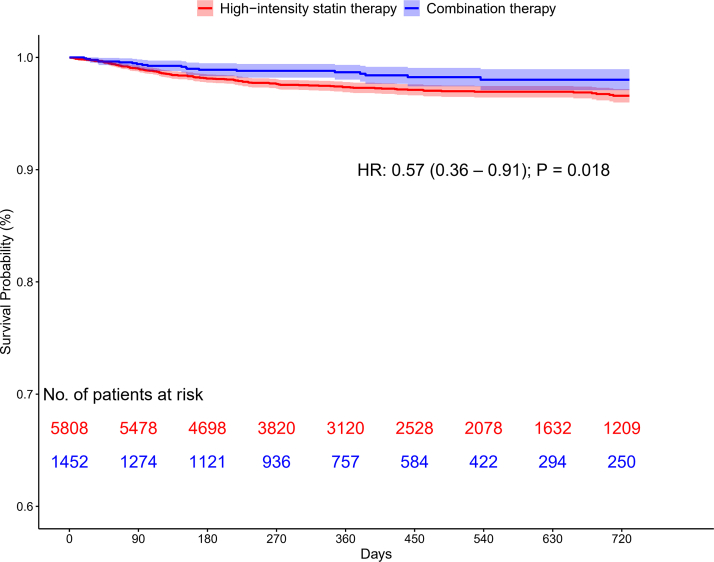
Kaplan–Meier curves for the incidence and 95% confidence intervals of PCI/CABG during follow-up in the propensity matched cohorts for those treated by high-intensity statin therapy or combination therapy.

Among 21,052 patients in the final sample, 1,452 patients received the combination therapy, and 19,600 patients received high-intensity therapy (Fig. 2). Before the propensity score matching, patients in the combination therapy group were more likely to have history of ischemic heart disease, and less likely to have history of stroke. Meanwhile, fewer patients in the combination therapy group were prescribed RAS inhibitors, and calcium channel blockers. Additionally, baseline LDL-C level was lower in the combination therapy group (Supplementary Table S5). After propensity score matching, 1,452 patients in the combination therapy group were matched to 5,808 patients in the high-intensity statin therapy group at a ratio of 1:4. All the baseline characteristics were well balanced with SMD less than 0.1. The mean age was 66.3 (9.7) years for the combination therapy group, and 66.2 (9.5) years for the high-intensity statin therapy group (Table 1).

**Table 1:** Baseline characteristics after the propensity score matching.

	After the propensity score matching		
	High-intensity statin therapy (n = 5,808)	Combination therapy (n = 1,452)	SMD
Age (mean (SD))	66.2 (9.5)	66.3 (9.7)	0.016
Male (%)	3349 (57.7)	837 (57.6)	<0.001
LDL-C (mmol/L, mean (SD))	2.40 (0.58)	2.40 (0.74)	0.010
HCL-C (mmol/L, mean (SD))	1.37 (0.35)	1.36 (0.35)	0.023
TG (mmol/L, mean (SD))	1.31 (0.75)	1.32 (0.78)	0.020
Calendar year of index date (%)			0.036
2014	16 (0.3)	5 (0.3)	
2015	54 (0.9)	11 (0.8)	
2016	101 (1.7)	21 (1.4)	
2017	76 (1.3)	21 (1.4)	
2018	100 (1.7)	26 (1.8)	
2019	246 (4.2)	61 (4.2)	
2020	226 (3.9)	58 (4.0)	
2021	483 (8.3)	123 (8.5)	
2022	780 (13.4)	193 (13.3)	
2023	2339 (40.3)	587 (40.4)	
2024	1387 (23.9)	346 (23.8)	
Duration of moderate statin therapy (days, mean (SD))	703 (662)	703 (682)	0.001
Obesity (%)	671 (11.6)	180 (12.4)	0.026
Hypertension (%)	3409 (58.7)	862 (59.4)	0.014
Diabetes (%)	1358 (23.4)	341 (23.5)	0.002
Liver disease (%)	18 (0.3)	5 (0.3)	0.006
Chronic kidney disease (%)	168 (2.9)	45 (3.1)	0.012
Ischemic heart disease (%)	4192 (72.2)	1051 (72.4)	0.005
Peripheral vascular disease (%)	210 (3.6)	56 (3.9)	0.013
Stroke (%)	1609 (27.7)	399 (27.5)	0.005
Antiplatelet (%)	1621 (27.9)	407 (28.0)	0.003
Renin-Angiotensin-Aldosterone System inhibitors (%)	2357 (40.6)	591 (40.7)	0.002
Beta blockers (%)	2779 (47.8)	704 (48.5)	0.013
Calcium channel blockers (%)	4738 (81.6)	1182 (81.4)	0.004

Notes: LDL-C: low-density lipoprotein cholesterol, HDL-C: high-density lipoprotein cholesterol, TG: triglycerides.

### Combination therapy versus high-intensity statin therapy with incident PCI/CABG and MACE

The median follow-up time was 379 (202, 572) days for the combination therapy group, and 517 (244, 730) days for the high-intensity statin therapy group. During the 2-year follow-up, 20 patients (1.31 per 100 patient-years) in the combination therapy group received PCI/CABG, irrespective of propensity score matching; 551 patients (2.16 per 100 patient-years) and 149 (2.25 per 100 patient-years) patients in the high-intensity statin therapy group received PCI/CABG, before and after propensity score matching, respectively (Table 2). Fig. 2 showed the Kaplan–Meier plot of the incidence of PCI/CABG during the 2-year follow-up for the matched cohort. In the matched cohort, the combination therapy group had a significantly lower risk of PCI/CABG during the follow-up, with a hazard ratio (HR, 95% confidence interval [CI]) of 0.57 (95% CI: 0.36, 0.91). Additionally, the combination therapy group was also associated with a significantly lower risk of PCI/CABG within one year after the index date (HR, 0.51; 95% CI: 0.30, 0.85; Supplementary Fig. S2 and Table S6), as well as a reduced risk of MACE during the 2-year follow-up period (HR, 0.62; 95% CI: 0.45, 0.86; Supplementary Fig. S3 and Table S7).

**Table 2:** Association of the combination therapy with incident PCI/CABG during the follow-up.

	Number of Patients	Number of Events	Median Follow-up^a^	Incidence Rate^b^	HR (95% CI)
Unadjusted					
High-intensity statin therapy	21,052	551	517 (244, 730)	2.16 (1.98, 2.35)	Reference
Combination therapy	1,452	20	379 (202, 572)	1.31 (0.80, 2.02)	0.55 (0.35, 0.86)
Adjusted by PS matching					
High-intensity statin therapy	5,808	149	393 (216, 669)	2.25 (1.90, 2.64)	Reference
Combination therapy	1,452	20	379 (202, 572)	1.31 (0.80, 2.02)	0.57 (0.36, 0.91)

Notes: PS: propensity score, HR: hazard ratio, CI: confidence interval.

a Median follow-up is reported in days, with the interquartile range (IQR).

b Incidence rate is calculated as a number of new events per 100 person-years, with 95% confidence intervals.

### Change of LDL-C level in follow-up

In the matched cohort, 1,270 patients receiving the combination therapy, and 5,462 patients receiving high-intensity therapy, who had at least one LDL-C test record during the follow-up, were used for inter-group comparison for the follow-up change of LDL-C level. For the combination therapy group and the high-intensity statin therapy group, Table 3 presented the mean absolute and relative changes (including inter-group differences) in LDL-C level, as well as the percentages of patients whose LDL-C level was reduced to guideline-recommended target level. Compared to the high-intensity statin therapy group, the combination therapy group had a significant additional reduction of LDL-C level across the follow-up subintervals. Furthermore, the combination therapy group had a higher ratio of achieving the target LDL-C levels, including 1.8 mmol/L and 1.4 mmol/L (Table 3).

**Table 3:** Changes in LDL-C level during the follow-up in the matched cohort.

	30–90 days	90–180 days	180–365 days	365–545 days	545–730 days
Number of patients					
Combination therapy	419	755	711	424	241
High-intensity statin therapy	1,433	3,653	2,968	1,888	1,233
Absolute change (mmol/L)					
Combination therapy (mean [SD])	−0.74 (0.81)	−0.68 (0.66)	−0.66 (0.64)	−0.64 (0.74)	−0.75 (0.74)
High-intensity statin therapy (mean [SD])	−0.43 (0.62)	−0.38 (0.61)	−0.42 (0.62)	−0.46 (0.70)	−0.46 (0.70)
Inter-group difference (95% CI)	−0.31 (−0.39, −0.22)	−0.30 (−0.35, −0.25)	−0.24 (−0.30, −0.19)	−0.18 (−0.25, −0.10)	−0.29 (−0.40, −0.19)
P-value	<0.0001	<0.0001	<0.0001	<0.0001	<0.0001
Relative change (%)					
Combination therapy (mean [SD])	−27.8 (26.2)	−26.7 (23.4)	−26.3 (23.4)	−24.6 (26.9)	−28.1 (24.7)
High-intensity statin therapy (mean [SD])	−16.4 (22.6)	−14.7 (22.1)	−16.0 (22.7)	−17.4 (25.7)	−17.5 (25.8)
Inter-group difference (95% CI)	−11.4 (−14.1, −8.6)	−12.0 (−13.8, −10.1)	−10.3 (−12.2, −8.4)	−7.2 (−10.0, −4.4)	−10.6 (−14.1, −7.2)
P-value	<0.0001	<0.0001	<0.0001	<0.0001	<0.0001
Number/percentage of patients achieving 1.8 mmol/L (%)					
Combination therapy (n = 1,270)	966/76.1				
High-intensity statin therapy (n = 5,452)	2,879/52.8				
P-value	<0.0001				
Number/percentage of patients achieving 1.4 mmol/L (%)					
Combination therapy (n = 1,270)	527/41.5				
High-intensity statin therapy (n = 5,452)	989/18.1				
P-value	<0.0001				

Note: CI: confidence interval.

The high-intensity statin therapy group served as the reference group.

### Sensitivity analyses

Using two relaxed gap definitions (7 days and 14 days), 6,454 and 6,119 patients were excluded, respectively. After the propensity score matching, the association between combination therapy and incident PCI/CABG remained robust (Supplementary Table S8).

### Subgroup analysis

In the matched cohort, 1,070 patients receiving the combination therapy, and 4,315 patients receiving high-intensity therapy, whose baseline LDL-C level less than 2.6 mmol/L, were included for the subgroup analysis. In the 2-year follow-up, the combination therapy group had a significantly lower risk of PCI/CABG, with a HR of 0.51 (95% CI: 0.29, 0.91) (Supplementary Table S9). The results were consistent with the main analysis.

## Discussion

This study evaluated the association between adding ezetimibe versus switching to the high-intensity statin and the risk of PCI/CABG among patients with ASCVD receiving moderate-intensity statin therapy. Compared to the high-intensity statin therapy, the combination therapy was associated with a lower risk of first PCI/CABG. Furthermore, patients with ASCVD receiving the combination therapy had a significant additional reduction in LDL-C level during the follow-up.

LDL-C is a well-established and independent etiological factor in the pathogenesis of ASCVD. Previous studies have demonstrated a linear relationship between reductions in LDL-C levels and decrease in atherosclerotic plaque volume, which in turn are associated with significant reductions in major adverse cardiovascular events.^30^ Accordingly, current clinical guidelines recommend reducing LDL-C level of patients with ASCVD below 1.8 mmol/L, and even below 1.4 mmol/L for those at very high risk, primarily through statin therapy.^31^^,^^32^ Despite strong endorsement of high-intensity statins, some limitations should be noticed. First, with respect to the adherence to high-intensity statins, previous research indicated that only about 30%–50% of eligible patients are treated accordingly, a treatment gap that persists even in populations with good healthcare access and adherence.^33^ Secondly, in Asian population, clinicians may be more hesitant to prescribe high-dose statins, where lower tolerance to high-dose statins has been observed.^20^, ^21^, ^22^, ^23^ This tendency to prescribe moderate-intensity statins initially and maintain the dosage may contribute to suboptimal LDL-C lowering and residual cardiovascular risk.^34^^,^^35^

Ezetimibe is recommended for patients who do not reach LDL-C targets with statins alone. Beyond its cholesterol-lowering effects, ezetimibe may exert pleiotropic actions, including modulation of inflammatory pathways, oxidative stress, and vascular repair mechanisms.^36^^,^^37^ Compared with high-intensity statin monotherapy, the RACING trial demonstrated potential that adding ezetimibe to moderate-intensity statins yielded greater reductions in LDL-C level and was no-inferior in major adverse cardiovascular events (MACE), compared to high-intensity statin monotherapy.^16^ This trial did not show a significant reduction in the need for coronary revascularization (including PCI and CABG), highlighting the complexity of translating lipid improvements into procedural risk reduction. Further, recent cohort studies have suggested that combination therapy had additional benefits, including reducing the risk of repeat PCI and CABG, stroke, and myocardial infarction.^14^^,^^15^^,^^19^^,^^38^^,^^39^ Notably, previous studies included a high proportion of patients with prior PCI, and did not demonstrate an effect of combination therapy in reducing the risk of PCI or CABG within the first year of follow-up. While the addition of ezetimibe to moderate-intensity statins appears to confer incremental cardiovascular benefits over high-intensity statin monotherapy in the previous randomised controlled trials, the early benefit of combination therapy was not observed. These findings underscore the need to identify the optimal timing, specific patient populations, and precise clinical scenarios for initiating combination therapy.

Compared with the RACING trial and other previous studies that with a high proportion of patients with prior PCI,^14^, ^15^, ^16^^,^^19^^,^^38^, ^39^, ^40^ our study explored the early benefits of adding ezetimibe instead of using the high-intensity statin therapy, to patients without history of coronary revascularization, treated with moderate-intensity statin therapy, and required to strength the intensity of lipid-lowering therapy. These early benefits may be explained by differences in both patient characteristics and the dynamics of LDL-C reduction. This study excluded individuals with previous coronary revascularization, making early events more likely driven by de novo plaque progression rather than procedure-related factors that are less responsive to short-term lipid lowering.^41^, ^42^, ^43^, ^44^, ^45^ In addition, combination therapy achieved a more rapid and greater early reduction in LDL-C, facilitating earlier attainment of guideline-recommended targets and plaque stabilization.^45^^,^^46^ As the association between achieved LDL-C levels and PCI/CABG risk was similar across groups, the higher proportion of patients reaching LDL-C targets in the combination therapy group likely contributed to the lower early rates of PCI/CABG and MACE. In summary, the early benefit of combination therapy may delay the need for coronary revascularization and achieve greater LDL-C reduction, thereby providing a clinically meaningful therapeutic window for subsequent ASCVD management.

To our knowledge, this is the first observational study using real-world data to evaluate the effectiveness of combination therapy on PCI/CABG incidence and LDL-C reduction in Chinese patients with ASCVD receiving moderate-intensity statin therapy. For the Asian population, especially Chinese population, who are at higher risk of using high-intensity statin, we found the combination therapy decreased the risk of incident PCI and CABG. Additionally, we measured the change in LCL-C level during the period of receiving combination therapy, which showed an additional reduction in both compared to the use of high-intensity statins, with a higher ratio of achieving the target LDL-C level.

Several limitations should be acknowledged. First, due to data constraints, the follow-up duration was limited to two years, which precludes assessment of long-term outcomes. However, the majority of cardiovascular events occurred within the first year, suggesting that most relevant outcomes were captured. Second, missing laboratory data increased over time, potentially affecting the accuracy of lipid profile measurements; nonetheless, the percentage change in LDL-C remained consistent across different intervals. Finally, in this retrospective study, residual confounding from unmeasured factors, such as medication adherence, lifestyle modifications, and socioeconomic status, may influence the results; however, these data were not available in CDARS.

In conclusion, using real-world clinical data, our study supports the potential early benefits of using the combination therapy of moderate-intensity statins and ezetimibe in high-risk Asian patients with ASCVD. This approach may reduce the risk of initial PCI/CABG and further lower the LDL-C level, providing an opportunity for further optimisation of ASCVD management. Clinicians may consider the initiation of combination therapy to personalize treatment strategies and improve cardiovascular outcomes. Future prospective randomized controlled trials are warranted to confirm these findings, and to evaluate both the early and long-term benefits of combination therapy.

## Contributors

HH, JT, VKCY, HFT, and EWC conceptualized the study. EWC provided resources, supervised the study, and acquired the data. HH, JT conducted the statistical analysis and verified the data. HH, JT, and EWC had access to raw data. HH drafted the manuscript. JEB, ECW, MTT and HFT provided clinical insights and suggestions. HH, JT, VKCY, JEB, ECM, MTT, HEY, HFT, and EWC contributed to the interpretation of the analysis, critically reviewed and revised the manuscript. HH, JT, VKCY, JEB, ECM, MTT, HEY, HFT, and EWC have read and approved the final version of the manuscript. EWC and HFT had final responsibility for the decision to submit for publication.

## Data sharing statement

Data are not available as the data custodians (the Hospital Authority and the Department of Health of Hong Kong SAR) have not given permission for sharing due to patient confidentiality and privacy consideration. Local academic institutions, government departments, or non-governmental organizations may apply for the access to data through the Hospital Authority’s data sharing portal (https://www3.ha.org.hk/data). All the analysis codes support the findings are available from the corresponding author upon reasonable requests.

## Editor note

The Lancet Group takes a neutral position with respect to territorial claims in published maps and institutional affiliations.

## Declaration of interests

Prof Esther W.Y. Chan has received grants from the National Natural Science Foundation of China; grants from Research Grants Council (RGC, HKSAR), Research Fund Secretariat of the Food and Health Bureau (Health and Medical Research Fund [HMRF], HKSAR), National Health and Medical Research Council (Australia), Narcotics Division of the Security Bureau of HKSAR, Amgen, AstraZeneca, Bayer, Bristol Myers Squibb, Eisai, Janssen, Pfizer, Takeda, and Novartis; and honorarium from Pfizer, Novartis, and the Hong Kong SAR Hospital Authority outside the submitted work. Dr. Joseph Edgar Blais reported receiving research grants from the Research Fund Secretariat of the Food and Health Bureau (Health and Medical Research Fund [HMRF], Hong Kong SAR) and consulting fees from the Institute of Medical Advancement and Clinical Excellence (IMACE) Hong Kong, outside the submitted work. Dr. Hei Hang Edmund Yiu reported receiving research grants from the Health Bureau of the Government of the Hong Kong SAR (HMRF) and Viatris, and an honorarium from the Southern Pharmacoeconomics Forum, outside the submitted work.

No other disclosures were reported.

## References

[1] Mousavi I., Suffredini J., Virani S.S. (2024). Early-onset atherosclerotic cardiovascular disease. Eur J Prev Cardiol.

[2] Nedkoff L., Briffa T., Zemedikun D., Herrington S., Wright F.L. (2023). Global trends in atherosclerotic cardiovascular disease. Clin Ther.

[3] Zhang X., Chen Z., Fang A. (2024). Trends in prevalence, risk factor control and medications in atherosclerotic cardiovascular disease among US Adults, 1999–2018. Am J Prev Cardiol.

[4] Alkhouli M., Alqahtani F., Kalra A. (2020). Trends in characteristics and outcomes of hospital inpatients undergoing coronary revascularization in the United States, 2003-2016. JAMA Netw Open.

[5] Lawton J.S., Tamis-Holland J.E., Bangalore S. (2022). 2021 ACC/AHA/SCAI guideline for coronary artery revascularization: a report of the American college of Cardiology/American Heart Association Joint committee on clinical practice guidelines. Circulation.

[6] Rao S.V., O’Donoghue M.L., Ruel M. (2025). 2025 ACC/AHA/ACEP/NAEMSP/SCAI guideline for the management of patients with acute coronary syndromes: a report of the American college of Cardiology/American Heart Association Joint Committee on clinical practice Guidelines. Circulation.

[7] Gaudino M., Andreotti F., Kimura T. (2023). Current concepts in coronary artery revascularisation. Lancet.

[8] Gu D., Qu J., Zhang H., Zheng Z., Wang M. (2020). Coronary artery disease: therapeutics and drug discovery.

[9] Lawton J.S., Tamis-Holland J.E., Bangalore S. (2022). 2021 ACC/AHA/SCAI Guideline for coronary artery revascularization: executive summary: a report of the American college of Cardiology/American Heart Association Joint Committee on clinical practice Guidelines. Circulation.

[10] Voudris K.V., Kavinsky C.J. (2019). Advances in management of stable coronary artery disease: the role of revascularization?. Curr Treat Options Cardiovasc Med.

[11] Virani S.S., Newby L.K., Arnold S.V. (2023). 2023 AHA/ACC/ACCP/ASPC/NLA/PCNA Guideline for the management of patients with chronic coronary disease: a report of the American heart Association/American College of Cardiology Joint Committee on clinical practice guidelines. Circulation.

[12] Vrints C., Andreotti F., Koskinas K.C. (2024). 2024 ESC guidelines for the management of chronic coronary syndromes: developed by the task force for the management of chronic coronary syndromes of the European Society of Cardiology (ESC) endorsed by the European Association for Cardio-Thoracic Surgery (EACTS). Eur Heart J.

[13] Grundy S.M., Stone N.J., Bailey A.L. (2019). 2018 AHA/ACC/AACVPR/AAPA/ABC/ACPM/ADA/AGS/APhA/ASPC/NLA/PCNA guideline on the management of blood cholesterol: executive summary: a report of the American College of Cardiology/American Heart Association task force on clinical practice Guidelines. Circulation.

[14] Leosdottir M., Schubert J., Brandts J. (2025). Early ezetimibe initiation after myocardial infarction protects against later cardiovascular outcomes in the SWEDEHEART registry. J Am Coll Cardiol.

[15] Lee S.-J., Joo Jae H., Park S. (2023). Combination lipid-lowering therapy in patients undergoing percutaneous coronary intervention. J Am Coll Cardiol.

[16] Kim B.-K., Hong S.-J., Lee Y.-J. (2022). Long-term efficacy and safety of moderate-intensity Statin with ezetimibe combination therapy versus high-intensity statin monotherapy in patients with atherosclerotic cardiovascular disease (RACING): a randomised, open-label, non-inferiority trial. Lancet.

[17] Cannon Christopher P., Blazing Michael A., Giugliano Robert P. (2015). Ezetimibe added to statin therapy after acute coronary syndromes. N Engl J Med.

[18] Mach F., Koskinas K.C., Roeters van Lennep J.E. (2025). 2025 focused update of the 2019 ESC/EAS guidelines for the management of dyslipidaemias. Atherosclerosis.

[19] Lee Y.-J., Hong B.-K., Yun K.H. (2025). Alternative LDL cholesterol–lowering strategy vs high-intensity statins in atherosclerotic cardiovascular disease: a systematic review and individual patient data meta-analysis. JAMA Cardiol.

[20] Fitchett D.H., Hegele R.A., Verma S. (2015). Statin intolerance. Circulation.

[21] Li Y.-F., Feng Q.-Z., Gao W.-Q., Zhang X.-J., Huang Y., Chen Y.-D. (2015). The difference between Asian and Western in the effect of LDL-C lowering therapy on coronary atherosclerotic plaque: a meta-analysis report. BMC Cardiovasc Disord.

[22] Birmingham B.K., Bujac S.R., Elsby R. (2015). Rosuvastatin pharmacokinetics and pharmacogenetics in caucasian and Asian subjects residing in the United States. Eur J Clin Pharmacol.

[23] Birmingham B.K., Bujac S.R., Elsby R. (2015). Impact of ABCG2 and SLCO1B1 polymorphisms on pharmacokinetics of rosuvastatin, atorvastatin and simvastatin acid in caucasian and Asian subjects: a class effect?. Eur J Clin Pharmacol.

[24] Hong Kong hospital authority: introduction. https://www.ha.org.hk/visitor/ha_index.asp.

[25] Leung W.K., Wong I.O.L., Cheung K.S. (2018). Effects of Helicobacter pylori treatment on incidence of gastric cancer in older individuals. Gastroenterology.

[26] Lau W.C.Y., Chan E.W., Cheung C.-L. (2017). Association between dabigatran vs warfarin and risk of osteoporotic fractures among patients with nonvalvular atrial fibrillation. JAMA.

[27] Cheung K.S., Chan E.W., Wong A.Y.S., Chen L., Wong I.C.K., Leung W.K. (2018). Long-term proton pump inhibitors and risk of gastric cancer development after treatment for helicobacter pylori: a population-based study. Gut.

[28] Haukoos J.S., Lewis R.J. (2015). The propensity score. JAMA.

[29] Rassen J.A., Shelat A.A., Myers J., Glynn R.J., Rothman K.J., Schneeweiss S. (2012). One-to-many propensity score matching in cohort studies. Pharmacoepidemiol Drug Saf.

[30] Cholesterol Treatment Trialists’ (CTT) Collaboration (2010). Efficacy and safety of more intensive lowering of LDL cholesterol: a meta-analysis of data from 170 000 participants in 26 randomised trials. Lancet.

[31] Mach F., Baigent C., Catapano A.L. (2019). 2019 ESC/EAS guidelines for the management of dyslipidaemias: lipid modification to reduce cardiovascular risk: the task force for the management of dyslipidaemias of the European Society of Cardiology (ESC) and European Atherosclerosis Society (EAS). Eur Heart J.

[32] Grundy S.M., Stone N.J., Bailey A.L. (2019). 2018 AHA/ACC/AACVPR/AAPA/ABC/ACPM/ADA/AGS/APhA/ASPC/NLA/PCNA guideline on the management of blood cholesterol: a report of the American College of Cardiology/American Heart Association Task Force on Clinical Practice Guidelines. J Am Coll Cardiol.

[33] Nelson Adam J., Haynes K., Shambhu S. (2022). High-intensity statin use among patients with atherosclerosis in the U.S. J Am Coll Cardiol.

[34] Lu Y., Zhang H., Lu J. (2021). Prevalence of dyslipidemia and availability of lipid-lowering medications among primary health care settings in China. JAMA Netw Open.

[35] Bi L., Yi J., Wu C. (2022). Atherosclerotic cardiovascular disease risk and lipid-lowering therapy requirement in China. Front Cardiovasc Med.

[36] Tsujita K., Sugiyama S., Sumida H. (2015). Impact of dual lipid-lowering strategy with ezetimibe and atorvastatin on coronary plaque regression in patients with percutaneous coronary intervention. J Am Coll Cardiol.

[37] Crea F., Niccoli G. (2015). Ezetimibe and plaque regression∗. J Am Coll Cardiol.

[38] Jun J.E., Jeong I.-K., Ahn K.J., Chung H.Y., Hwang Y.-C. (2024). Combination of low- or moderate-intensity statin and ezetimibe vs. high-intensity statin monotherapy on primary prevention of cardiovascular disease and all-cause death: a propensity-matched nationwide cohort study. Eur J Prev Cardiol.

[39] Lee S.-H., Lee Y.-J., Heo J.H. (2023). Combination moderate-intensity Statin and ezetimibe therapy for elderly patients with atherosclerosis. J Am Coll Cardiol.

[40] Lee S.-J., Joo J.H., Park S. (2023). Combination therapy with moderate-intensity atorvastatin and ezetimibe vs. high-intensity atorvastatin monotherapy in patients treated with percutaneous coronary intervention in practice: assessing RACING generalizability. Eur Heart J Cardiovasc Pharmacother.

[41] Zhang H., Zhang C., Zhang Y. (2024). The role of residual inflammatory risk and LDL cholesterol in patients with in-stent restenosis undergoing percutaneous coronary intervention. J Clin Lipidol.

[42] Kjøller-Hansen L., Maehara A., Kelbæk H. (2024). Impact of lipidic plaque on In-Stent and stent edge–related events after PCI in myocardial infarction: a PROSPECT II substudy. Circ Cardiovasc Interv.

[43] Angiolillo D.J., Galli M., Alexopoulos D. (2024). International consensus statement on platelet function and genetic testing in percutaneous coronary intervention: 2024 update. Cardiovasc Interv.

[44] Fujisaki T., Shirahama Y., Sheng F. (2025). The effect of lipid-lowering therapy on coronary artery plaque in East Asia population. JACC Asia.

[45] Banach M., Surma S., Guzik T.J. (2025). Upfront lipid-lowering combination therapy in high cardiovascular risk patients: a route to effective atherosclerotic cardiovascular disease prevention. Cardiovasc Res.

[46] Hirai K., Kawasaki T., Soejima T. (2023). Impact of intensive low-density lipoprotein cholesterol-lowering therapy on coronary artery plaques in acute coronary syndrome. Am J Cardiol.

