# Supplementary material for: Combination therapy with moderate-intensity statins and ezetimibe and risk of incident PCI/CABG in atherosclerotic cardiovascular disease: a propensity-matched cohort study
Source: Lancet Reg Health West Pac. 2026 Jun 5;71:101895. doi: 10.1016/j.lanwpc.2026.101895 (PMC13264374; doi:10.1016/j.lanwpc.2026.101895)

Supplementary materials

Table S1. Definition of atherosclerotic cardiovascular disease (ASCVD)

ASCVD	ICD-9 Codes[1]
**Disease**	
Ischemic heart disease	410, 411. 412, 413, 414
Cerebrovascular disease	433, 434, 435, 436
Peripheral vascular disease	441, 442, 443
**Surgery**	
Percutaneous coronary intervention (PCI)	36.01, 36.02, 36.05-36.07
Coronary artery bypass graft surgery (CABG)	36.1, 36.2
Other coronary procedures	36.00, 36.03, 36.04, 36.09, 36.3, 36.9

Note:
1. The primary outcome was defined using the ICD-9 codes for PCI and CABG.

Table S2. Definition of major adverse cardiovascular events

	ICD-9 code	ICD-10 code
Myocardial infarction	410	/
Stroke	433.01, 433.11, 433.21, 433.31, 433.81, 433.91, 434, 436, 437.0, 437.1, 430, 431, 432	/
Cardiovascular death	/	I099, I10, I110, I119, I129, I139, I200, I21, I210, I214, I219, I233, I248, I249, I25, I251, I255, I256, I259, I269, I270, I309, I311, I312, I313, I330, I340, I341, I350, I351, I359, I38, I420, I421, I422, I424, I426, I428, I429, I438, I461, I469, I471, I472, I48, I490, I499, I50, I500, I501, I506, I509, I510, I516, I519, I60, I609, I613, I614, I619, I620, I629, I634, I639, I64, I679, I710, I711, I712, I713, I728, I739, I823, I99

Table S3. Definition of statin intensity

Statin	Moderate-Intensity Dosage (LDL-C Reduction 30% to <50%)	High-Intensity Dosage (LDL-C Reduction ⩾50%)
Simvastatin	20 to 40 mg	NA
Atorvastatin	10 to 20 mg	40 to 80 mg
Rosuvastatin	5 to 10 mg	20 to 40 mg

From ACC/AHA, 2018.[13] Daily dosages are shown in the table.

Notes:
ACC: American College of Cardiology
AHA: American Heart Association
LDL-C: Low-density lipoprotein cholesterol
NA: Not applicable

Table S4. Baseline comorbidities and medication use

Comorbidity disease	ICD-9 code
Obesity	278.0, V85.3, V85.4
Hypertension	401, 402,403, 404,405, 437.2
Diabetes	250
Liver disease	456.0, 456.1, 456.2, 571.2, 571.4, 571.5, 571.6. 572.2, 572.3, 572.4, 572.8
Chronic kidney disease	585
Ischemic heart disease	410, 411, 412, 413, 414
Peripheral vascular disease	440, 441, 442, 443
Stroke	433, 434, 435, 436
**Drug Class of Medication use**	**BNF code**
Antiplatelets	2.9
Renin-Angiotensin-Aldosterone System inhibitors	2.5.5.1, 2.5.5.2
Beta blockers	2.4
Calcium channel blockers	2.6.2

Notes:
ICD-9: the International Classification of Diseases, Ninth Revision.
BNF: British National Formulary.

Table S5. Baseline characteristics before the Propensity Score Matching

	**Before the Propensity Score Matching**		
	**High-intensity statin therapy (n = 19,600)**	**Combination therapy (n = 1,452)**	**SMD**
Age (mean (SD))	66.5 (9.8)	66.3 (9.7)	0.013
Male (%)	11862 (60.5)	837 (57.6)	0.059
LDL-C (mmol/L, mean (SD))	2.50 (0.67)	2.40 (0.74)	0.143
HCL-C (mmol/L, mean (SD))	1.34 (0.34)	1.36 (0.35)	0.061
TG (mmol/L, mean (SD))	1.36 (0.79)	1.32 (0.78)	0.041
Calendar year of index date (%)			0.555
2014	56 (0.3)	5 (0.3)	
2015	176 (0.9)	11 (0.8)	
2016	348 (1.8)	21 (1.4)	
2017	523 (2.7)	21 (1.4)	
2018	844 (4.3)	26 (1.8)	
2019	1219 (6.2)	61 (4.2)	
2020	1923 (9.8)	58 (4.0)	
2021	3136 (16.0)	123 (8.5)	
2022	3793 (19.4)	193 (13.3)	
2023	5139 (26.2)	587 (40.4)	
2024	2443 (12.5)	346 (23.8)	
Duration of moderate statin therapy (days, mean (SD))	736 (680)	703 (682)	0.048
Obesity (%)	2499 (12.8)	180 (12.4)	0.011
Hypertension (%)	12006 (61.3)	862 (59.4)	0.039
Diabetes (%)	5183 (26.4)	341 (23.5)	0.068
Liver disease (%)	35 (0.2)	5 (0.3)	0.032
Chronic kidney disease (%)	497 (2.5)	45 (3.1)	0.034
Ischemic heart disease (%)	12404 (63.3)	1051 (72.4)	0.196
Peripheral vascular disease (%)	692 (3.5)	56 (3.9)	0.017
Stroke (%)	7151 (36.5)	399 (27.5)	0.194
Antiplatelet (%)	4638 (23.7)	407 (28.0)	0.100
Renin-Angiotensin-Aldosterone System inhibitors (%)	9098 (46.4)	591 (40.7)	0.115
Beta blockers (%)	10102 (51.5)	704 (48.5)	0.061
Calcium channel blockers (%)	17225 (87.9)	1182 (81.4)	0.180

Notes:
LDL-C: low-density lipoprotein cholesterol
HDL-C: high-density lipoprotein cholesterol
TG: triglycerides

Table S6. Association of the combination therapy with incident PCI/CABG within one year after the index date.

	Number of Patients	Number of Events	Median Follow-up[1]	Incidence Rate[2]	HR (95% CI)
**Unadjusted**					
High-intensity statin therapy	21,052	470	365 (244, 365)	2.92 (2.66, 3.20)	Reference
Combination therapy	1,452	16	365 (202, 365)	1.46 (0.83, 2.37)	0.49 (0.30, 0.81)
**Adjusted by PS Matching**					
High-intensity statin therapy	5,808	133	365 (216, 365)	2.91 (2.43, 3.44)	Reference
Combination therapy	1,452	16	365 (202, 365)	1.46 (0.83, 2.37)	0.51 (0.30, 0.85)

Notes:
PS: propensity score
HR: hazard ratio.
CI: confidence interval.
1. Median follow-up is reported in days, with the interquartile range (IQR).
2. Incidence rate is calculated as a number of new events per 100 person-years, with 95% confidence intervals.

Table S7. Association of the combination therapy with MACE during the follow-up

	Number of Patients	Number of Events	Median Follow-up[1]	Incidence Rate[2]	HR (95% CI)
**Unadjusted**					
High-intensity statin therapy	21,052	1110	504 (236, 730)	4.40 (4.15, 4.67)	Reference
Combination therapy	1,452	44	377 (199, 572)	2.88 (2.10, 3.87)	0.61 (0.45, 0.82)
**Adjusted by PS Matching**					
High-intensity statin therapy	5,808	298	385 (211, 662)	4.56 (4.05, 5.10)	Reference
Combination therapy	1,452	44	377 (199, 572)	2.88 (2.10, 3.87)	0.62 (0.45, 0.86)

Notes:
PS: propensity score
HR: hazard ratio.
CI: confidence interval.
1. Median follow-up is reported in days, with the interquartile range (IQR).
2. Incidence rate is calculated as a number of new events per 100 person-years, with 95% confidence intervals.

Table S8. Sensitivity analyses—association of the combination therapy with incident PCI/CABG using two gap definitions (7 days and 14 days)

	Number of Patients	Number of Events	Median Follow-up[1]	Incidence Rate[2]	HR (95% CI)[3]
**Sensitivity analyses**					
**Gap definition: 7 days**					
High-intensity statin therapy	5,808	144	397 (222, 672)	2.15 (1.81, 2.53)	Reference
Combination therapy	1,452	20	379 (202, 572)	1.31 (0.80, 2.02)	0.60 (0.37, 0.95)
**Gap definition: 14 days**	5,808				
High-intensity statin therapy	5,808	146	398 (218, 671)	2.19 (1.85, 2.58)	Reference
Combination therapy	1,452	20	379 (202, 572)	1.31 (0.80, 2.02)	0.58 (0.36, 0.93)

Notes:
HR: hazard ratio.
CI: confidence interval.
1. Median follow-up is reported in days, with the interquartile range (IQR).
2. Incidence rate is calculated as a number of new events per 100 person-years, with 95% confidence intervals.
3. The analysis was performed after propensity score matching.

Table S9. Subgroup analysis—association of the combination therapy with incident PCI/CABG among patients with baseline LDL-C level lower than 2.6 mmol/L

	Number of Patients	Number of Events	Median Follow-up[1]	Incidence Rate[2]	HR (95% CI)[3]
**Subgroup analysis**					
High-intensity statin therapy	4,315	107	402 (224, 663)	2.15 (1.76, 2.59)	Reference
Combination therapy	1,070	13	391 (221, 572)	1.12 (0.59, 1.91)	0.51 (0.29, 0.91)

Notes:
HR: hazard ratio.
CI: confidence interval.
1. Median follow-up is reported in days, with the interquartile range (IQR).
2. Incidence rate is calculated as a number of new events per 100 person-years, with 95% confidence intervals.
3. The analysis was performed after propensity score matching.

Figure S1. Study design

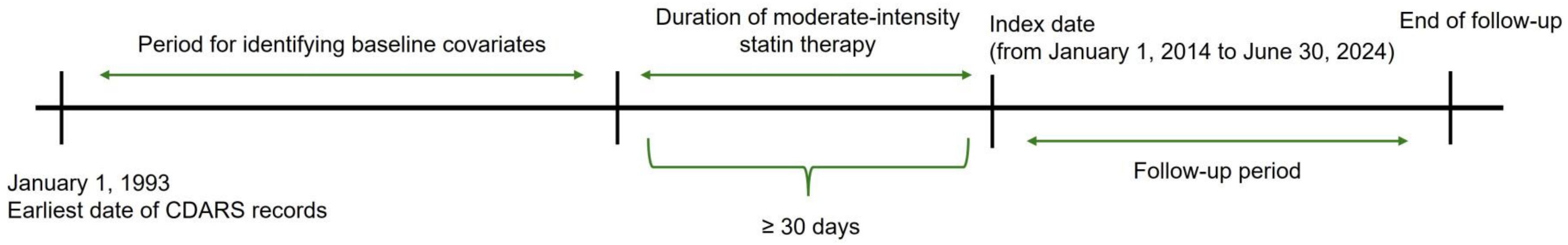

Figure S2. Kaplan-Meier curve for the incidence and 95% confidence intervals of PCI/CABG within one year after the index date in the propensity matched cohort for those treated by high-intensity statin therapy or combination therapy

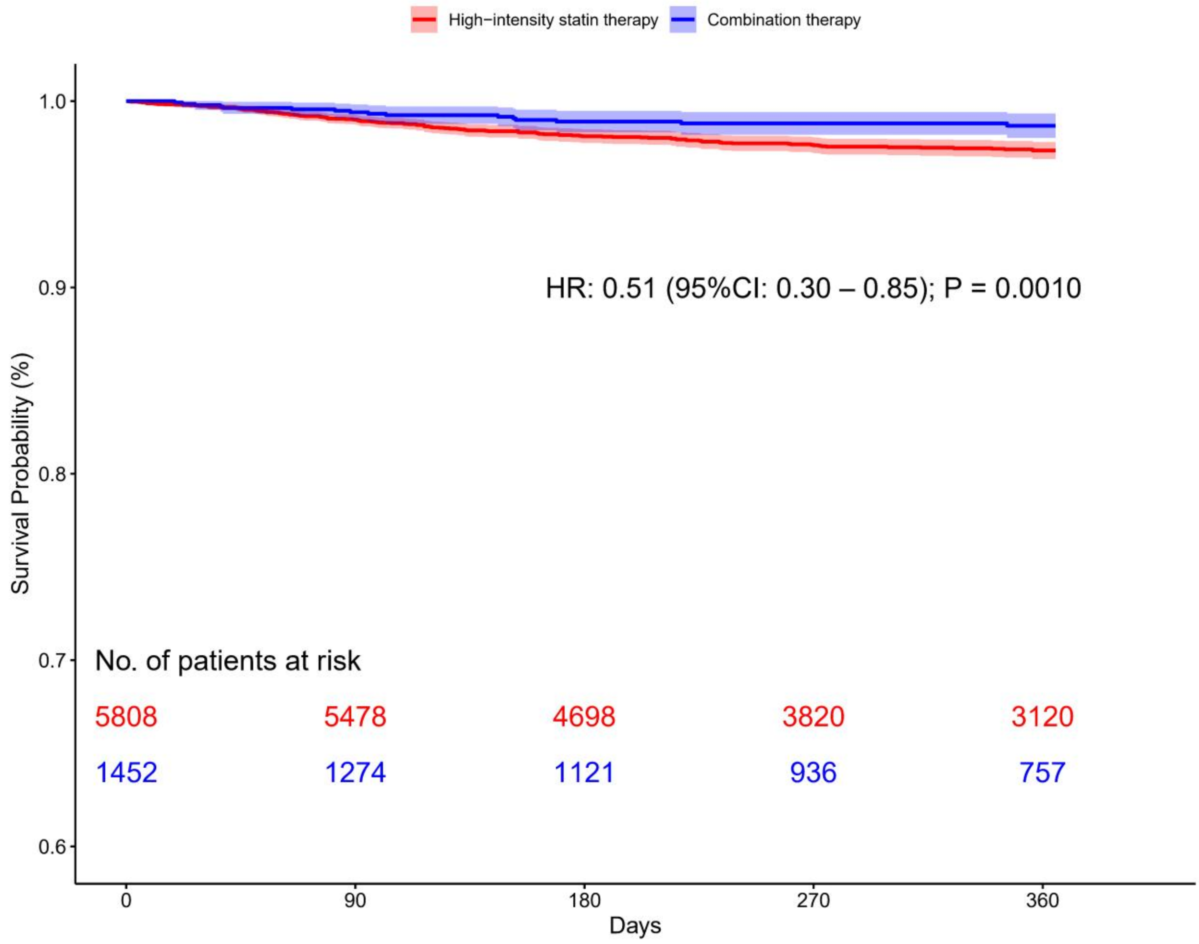

Figure S3. Kaplan-Meier curve for the incidence and 95% confidence intervals of MACE during follow-up in the propensity matched cohort for those treated by high-intensity statin therapy or combination therapy

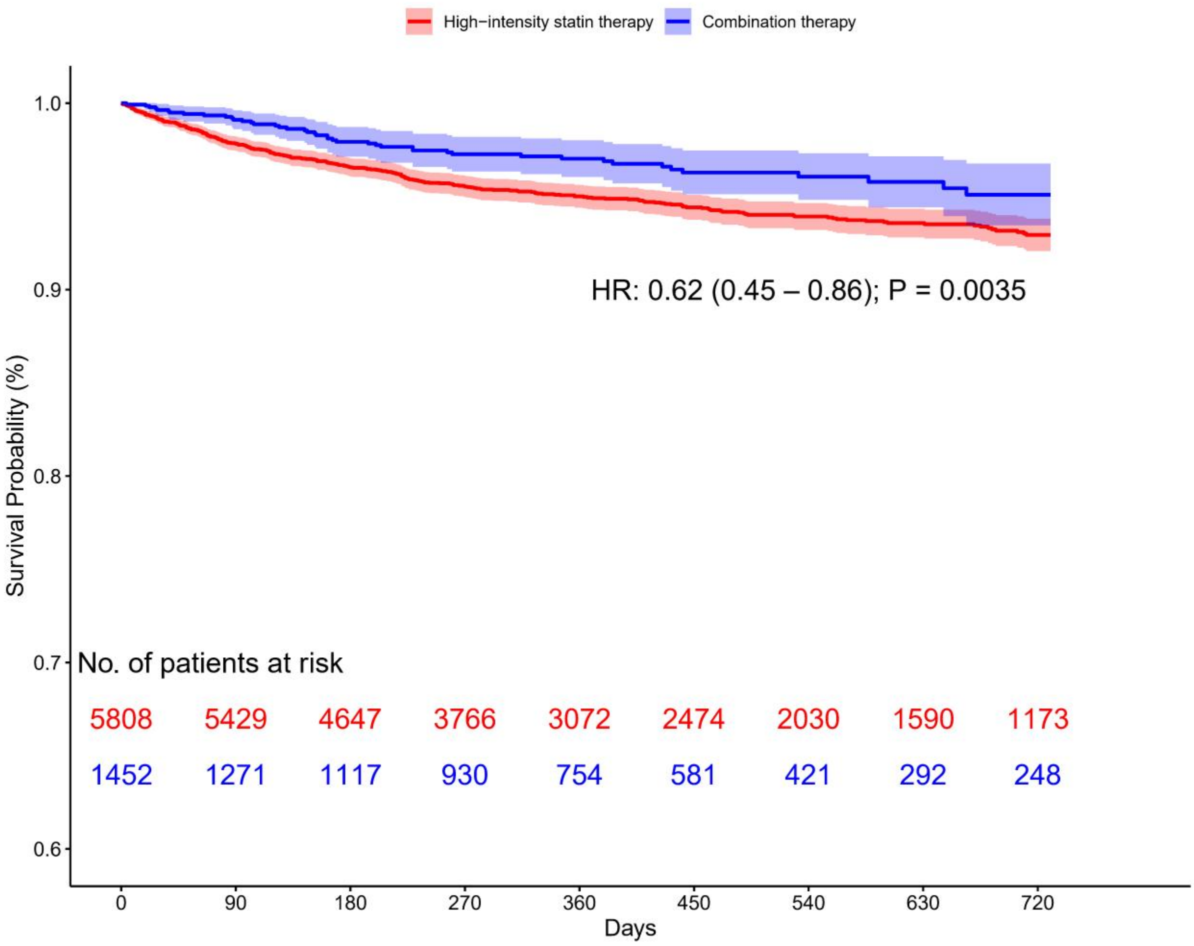

Supplement: Supplementary Figs. S1–S3 and Tables S1–S9 [file mmc1.pdf]
